# Enzymatic Polycondensation of 1,6-Hexanediol and Diethyl Adipate: A Statistical Approach Predicting the Key-Parameters in Solution and in Bulk

**DOI:** 10.3390/polym12091907

**Published:** 2020-08-24

**Authors:** Kifah Nasr, Julie Meimoun, Audrey Favrelle-Huret, Julien De Winter, Jean-Marie Raquez, Philippe Zinck

**Affiliations:** 1 University Lille, CNRS, Centrale Lille, Univ. Artois, UMR 8181—UCCS—Unité de Catalyse et Chimie du Solide, F-59000 Lille, France; kifah.nasr@univ-lille.fr (K.N.); julie.meimoun@univ-lille.fr (J.M.); audrey.huret@univ-lille.fr (A.F.-H.); 2 Laboratory of Polymeric and Composite Materials (LPCM), Center of Innovation and Research in Materials and Polymers (CIRMAP), University of Mons, Place du Parc 23, 7000 Mons, Belgium; 3 Center for Mass Spectrometry, Organic Synthesis and Mass Spectrometry Laboratory, University of Mons—UMONS, 23 Place du Parc, 7000 Mons, Belgium; Julien.dewinter@umons.ac.be

**Keywords:** enzymatic polymerization, polycondensation, lipase, polyesters, response surface methodology, recyclability

## Abstract

Among the various catalysts that can be used for polycondensation reactions, enzymes have been gaining interest for three decades, offering a green and eco-friendly platform towards the sustainable design of renewable polyesters. However, limitations imposed by their delicate nature, render them less addressed. As a case study, we compare herein bulk and solution polycondensation of 1,6-hexanediol and diethyl adipate catalyzed by an immobilized lipase from Candida antarctica. The influence of various parameters including time, temperature, enzyme loading, and vacuum was assessed in the frame of a two-step polymerization with the help of response surface methodology, a statistical technique that investigates relations between input and output variables. Results in solution (diphenyl ether) and bulk conditions showed that a two-hour reaction time was enough to allow adequate oligomer growth for the first step conducted under atmospheric pressure at 100 °C. The number-average molecular weight (*M_n_*) achieved varied between 5000 and 12,000 g·mol^−1^ after a 24 h reaction and up to 18,500 g∙mol^−1^ after 48 h. The statistical analysis showed that vacuum was the most influential factor affecting the *M_n_* in diphenyl ether. In sharp contrast, enzyme loading was found to be the most influential parameter in bulk conditions. Recyclability in bulk conditions showed a constant *M_n_* of the polyester over three cycles, while a 17% decrease was noticed in solution. The following work finally introduced a statistical approach that can adequately predict the *M_n_* of poly(hexylene adipate) based on the choice of parameter levels, providing a handy tool in the synthesis of polyesters where the control of molecular weight is of importance.

## 1. Introduction

Aliphatic and aromatic polyesters are more and more receiving special attention as biobased alternatives to petropolymers due to their vast availability, large range of applications, and to some of them, their biodegradability [1,2,3,4]. Their synthesis can be done either by the ring-opening polymerization of cyclic esters, or by polycondensation reactions. The conventional method for the polycondensation of aliphatic monomers uses metal-based catalysts and requires elevated temperatures [5,6,7]. However, reactions using metal-based catalysts can suffer from certain issues such as color changes, degradation due to high temperature, lack of selectivity and difficulty in the removal of residual metals from the synthesized polymer [8,9]. Accordingly, in recent years, there has been growing research on replacing metal-based catalysts with more eco-friendly organocatalysts and enzymes, both of which possessed certain advantages and limitations [10]. This work will focus on enzymatic catalysis as a replacement to conventional metal-based catalysts, due to their selectivity, being eco-friendly, and recyclable nature [5,10,11,12,13,14,15,16]. The most popular biocatalyst used is Novozym 435 (N435—lipase B from Candida Antarctica immobilized on an acrylic resin), where it showed improved properties in terms of specificity, thermal stability and selectivity, and was proposed in many research works as a versatile catalyst that can be beneficial in different synthetic routes, particularly in the case of aliphatic polyesters [17,18]. For instance, the large scale production of aliphatic polyesters via enzymatic catalysis was reported using adipic acid and 1,6-hexanediol as monomers and N435 as a catalyst [19]. Another polyester, poly(butylene succinate) was synthesized via enzymatic polycondensation of diethyl succinate and 1,4-butanediol both in organic solvent and in bulk conditions [20]. N435 was reported as a catalyst for the combination of ring-opening polymerization and polycondensation reactions using glycidol, ω-pentadecalactone and adipic acid as starting materials [21]. Similarly, ω-pentadecalactone, diethyl succinate and 1,4-butanediol were catalyzed via Candida antartica Lipase B (CALB) yielding a 77,000 g·mol^−1^ polyester [10,22]. Regarding its regioselectivity, Kulshrestha et al. [23] reported the biosynthesis (N435) of linear copolyesters starting with glycerol, 1,8-octanediol and adipic acid. The regioselectivity towards primary alcohol esterification was between 77% to 82%. Similarly, Zeng et al. showed that in the polycondensation of glycerol, 1,8-octanediol and adipic acid, N435 produced a close to linear polyester, where the selectivity of acylation at the primary hydroxyl sites of glycerol was 74.9%, and the number average molecular weight (*M_n_*) achieved was 22,700 g·mol^−1^. Those results were superior to other catalysts such as scandium triflate and organocatalysts such as 1,5,7-triazabicyclo [4,4,0]dec-5-ene (TBD), diphenyl hydrogen phosphate and bis(1,1,2,2,3,3,4,4,4-nonafluoro-1-butanesulfonyl)imide, which did not exceed a selectivity of 65.9% or yield molecular weights higher than 6000 g·mol^−1^ [24].

Many of the research in the area of enzymatic polyesterification remains very empirical, i.e., testing the viability and efficiency of different enzymes for the development of polyesters and optimizing the reaction conditions for achieving high molecular weights. However, the impact of certain parameters, or in a more accurate sense, the ‘degree of impact’ of these parameters can vary largely in different conditions and settings. Passing from solution to bulk remains very challenging and certain parameters, such as vacuum application, can behave differently. Although the importance of vacuum in the two-step method is very well established [5,25,26,27], most research included vacuum at a constant value, or otherwise compared it to reactions under atmospheric pressure. Poojari et al. [28] showed that an increase in vacuum gauge pressure from 66 to 400 mbar resulted in a significant increase in *M_n_* in the polycondensation reactions between 1,3-bis(3-carboxypropyl)tetramethyldisiloxane and 1,4-butanediol or 1,6-hexanediol. In the presence of 1,8-octanediol, the vacuum effect was reversed. Jiang [22] showed the necessity of high vacuum application to synthesize high molecular weight copolymers of ω-pentadecalactone, diethyl succinate and 1,4-butanediol by comparing the results achieved at high vacuum (<4 mbar) to very low levels of vacuum being ~ 800 and 1013 mbar (atmospheric pressure), respectively, where the two latter conditions produced oligomers that did not exceed 1000 g·mol^−1^ in *M_n_* compared to values exceeding 10,000 g·mol^−1^ at high vacuum levels.

Traditional methods of optimization follow a one-factor-at-a-time approach (OFAT), which involves varying one factor while keeping other factors constant. The main drawback of this method is that it does not account for interactions between the tested variables, leading to inaccuracy in depicting the true effect of the factors tested. On the other hand, the use of response surface methodology (RSM) such as central composite design (CCD) allows for an accurate representation of the effect of the tested variables and their interaction [29]. RSM has been successfully employed in many research works as a tool of optimization [30,31]. For example, Itabaiana et al. [32] used a CCD to determine optimal conditions for lipase catalyzed esterification of waste fatty acids into useful esters. Similarly, Pellis et al. [33] used a fractional factorial design to evaluate the effect of temperature, pressure and water content in the polycondensation reaction between 1,4-butanediol or 1,8-octanediol with dimethyl adipate catalyzed by either cutinase 1 from *Thermobifida cellulosilytica* or CALB, under solvent and thin film conditions.

In order to implement our response surface methodology, we select the step-growth synthesis of poly(hexylene adipate) conducted in solution and in bulk, following a two-step procedure, transesterification followed by polycondensation. After assessing the influence of oligomerization time and monomer concentration, the variable impact and interaction of different experimental parameters (temperature, % *w*/*w* enzyme loading and vacuum) on the polycondensation reaction between 1,6-hexanediol and diethyl adipate in solvent (diphenyl ether) and bulk media, using N435 is reported. Two central composite designs were used to build second-order quadratic models with equations that can predict the *M_n_* based on the conditions used. As such, these models give users the exact parameters that can be considered to develop a polymer with a certain desired range of *M_n_*, a method that can show to be very useful for providing an efficient tool in the enzymatic synthesis of polyesters using a step-growth method, where the control of *M_n_* is of importance. Finally, the influence of the process, bulk vs. solution, on enzyme recycling was also studied.

## 2. Materials and Methods

### 2.1. Materials

1,6-hexandiol (97%), diethyl adipate (99%) and diphenyl ether (99%) were purchased from Sigma-Aldrich (Lyon, France). Analytical grade methanol, tetrahydrofuran (THF), chloroform (99%) and toluene were purchased from VWR (Fontenay-sous-Bois, France). All the reagents and solvents were used as received. Novozym 435 (N435), a Candida antartica Lipase B (CALB) immobilized on an acrylic resin was kindly provided by Novozymes (Bagsværd, Denmark) (activity = 10,000 propyl laurate units (PLU)/g). Chloroform D (CDCl3) (99.8%) was purchased from Euriso-top (Saint-Aubin, France).

### 2.2. General Procedure of the Enzymatic Polycondensation of 1,6-Hexanediol and Diethyl Adipate

Equimolar amounts (4 mmol) of 1,6-hexanediol and diethyl adipate were weighed and added into a schlenk tube. A predetermined amount varied between 1% and 10% *w*/*w* of N435 (relative to the total weight of the monomers) was weighed and added to the mixture. For solution polymerization, 1 mL of diphenyl ether was added as a solvent of choice, whereas for bulk polymerization, the reaction was commenced with no additional steps. The reaction proceeded under atmospheric pressure for 2 h at a preset temperature between 80 and 100 °C (using an oil bath with continuous stirring kept constant at 350 rpm). Afterwards, the schlenk tube was attached to a vacuum line, and the pressure was decreased gradually in 1 h to reach a predetermined value between 10 and 50 mbar to remove byproduct (ethanol). After applying the vacuum, the reaction was left to proceed for 24 h and got stopped by adding an excess amount of chloroform under atmospheric pressure after a cooling step, followed by direct filtration to remove the N435 beads. The filtrate was then partially evaporated using a rotavap, and then added dropwise to excess amount of cold methanol under stirring to precipitate the obtained polymer. The mixture was then filtered, and the product obtained was left to dry at room temperature for 24 h before collecting and weighing. NMR spectra of 1,6-hexanediol, diethyl adipate and the precipitated poly(hexylene adipate) are provided in Figures S1–S3, respectively.

### 2.3. Effect of Solvent Volume on the Achieved Number-Average Molecular Weight (M_n_)

For solution polymerization, diphenyl ether was chosen as a solvent. To determine the effect of volume variation, the polycondensation reaction was carried out with different volumes at 100 °C at 1% *w*/*w* N435 for 2 h oligomerization followed by an additional 24 h polymerization under 10 mbar of vacuum (see Table 1). The reaction was then confirmed via ^1^H NMR analysis.

### 2.4. Effect of the Oligomerization Step Time on Conversion in Solution and Bulk

The conversion was monitored by exploiting the ^1^H NMR signals at *δ* = 4.11–4.14 of the methylene group (*CH_3_–CH_2_–O–*) of diethyl adipate (DEA) in addition to the signals at *δ* = 4.06 of the methylene (*–O–CH_2_–C_4_H_8_–CH_2_–O–*) of the poly(hexylene adipate). Conversion was calculated via the ratio of the signal representing poly(hexylene adipate) at *δ* = 4.06 relative to the summation of signals representing DEA and poly(hexylene adipate) at *δ* = 4.11–4.13 and at *δ* = 4.06, respectively (see Equation (S1) in supporting information). Examples of NMR spectra are presented in Figures S4 and S5.

### 2.5. Effect of the Oligomerization Step Time on the Achieved Number-Average Molecular Weight (M_n_) after 24 h of Post Vacuum Application in Bulk

Two-step polycondensation reactions were set up to run with a tunable oligomerization time (2, 4 and 6 h) at constant temperature (90 °C), % *w*/*w* enzyme loading (5.5%), and followed by a secondary 24 h step under vacuum (10 mbar) application. *M_n_* was determined by GPC analysis.

### 2.6. N435 Recyclability

The polycondensation protocol of 1,6-hexandiol and diethyl adipate was followed as mentioned before, the temperature was kept constant at 100 °C and the oligomerization step at 2 h. At the end of each polymerization cycle, the separated N435 beads were washed 3 times with excess chloroform, then left to dry at room temperature for 24 h under atmospheric pressure and reused for three consecutive cycles.

### 2.7. Analytical Methods

#### 2.7.1. H NMR Analysis

Approximately 5 mg of 1,6-hexanediol, diethyl adipate and the recovered poly(hexylene adipate) were directly dissolved in three NMR tubes containing 0.5 mL of CDCl_3_. The ^1^H NMR spectra of the monomers and the recovered polymer were recorded at room temperature on a Bruker Avance 300 instrument (Bruker, Billerica, MA, USA), (delay time = 3 s, number of scans = 32) at 300.13 MHz. Chemical shifts (ppm) were calibrated using the residual signal of CDCl_3_ at 7.26 ppm. Additionally, ^1^H NMR was used to confirm conversion (detailed in supporting information). Data acquisition and analysis were performed using the Bruker TopSpin 3.2.

#### 2.7.2. GPC Analysis

Gel permeation chromatography analysis was performed in THF as eluent (flow rate of 1 mL/min) at 40 °C using Alliance e2695 apparatus (Waters, Milford, MA, USA) with a sample concentration around 10–15 mg·mL^−1^. A refractive index detector Optilab T-rEX (WYATT Optilab T-Rex, Santa Barbara, CA, USA) was used as a detector, and a set of columns: HR1, HR2 and HR4 (Water Styragel, Milford, MA, USA) were utilized. The molecular weight calibration curve was obtained using monodisperse polystyrene standards.

#### 2.7.3. MALDI-MS Analysis

Positive-ion Matrix assisted LASER Desorption/Ionization-Mass Spectrometry (MALDI-MS) experiments were performed using a Waters QToF Premier mass spectrometer (Waters, Milford, MA, USA) equipped with a Nd:YAG laser operating at 355 nm (third harmonic) with a maximum output of 65 µJ delivered to the sample in 2.2 ns pulses at 50 Hz repeating rate. Time-of-flight mass analysis was performed in the reflectron mode at a resolution of about 10 k (*m*/*z* 569). All samples were analyzed using trans-2-[3-(4-tert-butylphenyl)-2-methylprop-2-enylidene]malononitrile (DCTB) as a matrix. Polymer samples synthesized in bulk as well as in solution conditions were dissolved in THF to obtain 1 mg·mL^−1^ solution. Additionally, 40 µL of 2 mg·mL^−1^ NaI solution in acetonitrile was added to the polymer solution.

### 2.8. Statistical Analysis

A face-centered central composite design (CCD) was used to optimize the polycondensation reaction of poly(hexylene adipate) in terms of the established number-average molecular weight (*M_n_*). A CCD is an experimental designed used to determine the effect and interaction of several factors and develop a response model. This design consists of 3 levels represented as (−1, 0, 1), where the center point (0) is replicated several times to determine variability and improve predictability. In the following work, 3 factors were tested at 3 levels being temperature (80, 90, 100 °C), % *w*/*w* enzyme loading (1%, 5.5%, 10%), vacuum (10, 30, 50 mbar). The models were developed and analyzed by Design-Expert 11^®^ purchased from RITME (Paris, France). Build information of the design model for in solution and bulk polymerization is provided in Tables S1 and S5, respectively. The models were confirmed by running additional experiments that fell within 95% PI (prediction interval) range (see Tables S4 and S8).

## 3. Results

**Scheme 1:**
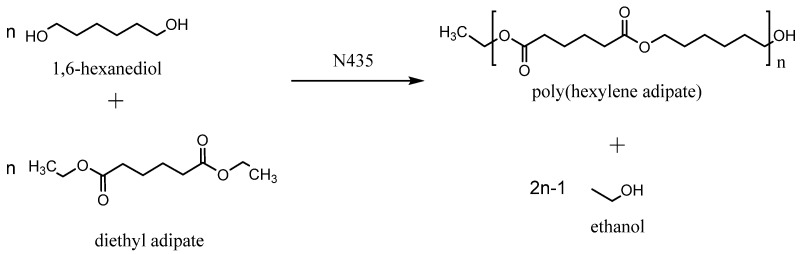
Polycondensation of 1,6-hexanediol and diethyl adipate in the presence of N435 as catalyst.

The solution polycondensation of equimolar amounts (4 mmol) of 1,6-hexanediol and diethyl adipate in the presence of N435 (see Scheme 1) was conducted in diphenyl ether, as it was reported to be the more suitable solvent to achieve higher molecular weights [20,34]. 

**Figure 1:**
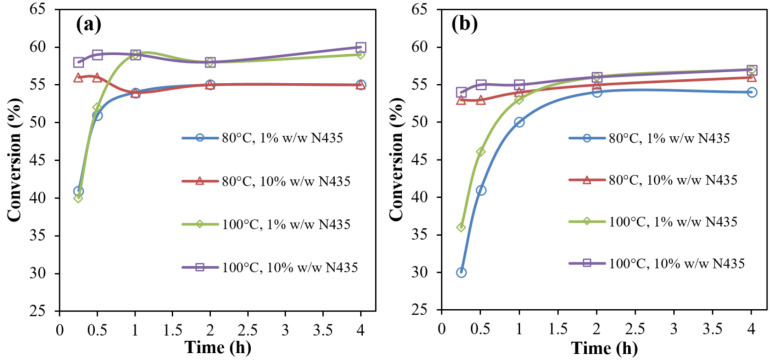
Conversion (expressed in %) after oligomerization step calculated via ^1^H NMR as a function of temperature and enzyme loading in bulk (**a**) and diphenyl ether (**b**) conditions.

The effect of the concentration on the yield and molecular weight was first assessed considering a 2 h oligomerization step followed by a 24 h polycondensation step under a vacuum of 10 mbar and a temperature of 100 °C. The overall concentrations were varied by changing the amount of diphenyl ether used. A typical ^1^H NMR spectrum is provided in the SI section (Figure S3), and the results are given in Table 1. An increase in *M_n_* and yield with monomer concentration can be noticed, e.g., where the *M_n_* passed from 7400 up to 12,300 g·mol^−1^ by increasing the monomer concentration from 0.5 to 4 mol·L^−1^. Similarly, the yield increased from 56% to 88%. Therefore, for the next experiments carried out in solution, the volume of diphenyl ether was set constant at 1 mL to establish a monomer concentration of 4 mol·L^−1^. This decrease in *M_n_* as a function of decreasing monomer concentration can be attributed to the decrease in the polymerization rate in dilute solutions due to the decrease in molecular collision as proposed by the collision theory [22,23]. In fact, this drop in reactivity is reflected as a decrease in monomer conversion, which would decrease the degree of polymerization (*X_n_*) according to Carothers equation: $X_{n}=1/(1-p)$, where *p* is defined as the conversion [35]. Another proposed cause can also be attributed to the decrease in the byproduct (ethanol) removal efficiency in lower concentration solutions [36]. As the vacuum application may result in the evaporation of small monomer fractions, the oligomerization step was varied between 1 and 6 h in both bulk and solution in order to determine a suitable time for oligomer growth before the vacuum application. 1,6-hexanediol and DEA were added in equimolar amounts (4 mmol) in both systems, while 1 mL of diphenyl ether was added for the solution polymerization method to obtain the previously optimized concentration (4 mol·L^−1^) conditions. The monomer conversion after the first step was determined via ^1^H NMR (see Figures S4 and S5) at different time intervals in order to assess the reaction kinetics and the time needed to reach the equilibrium at different temperatures and % enzyme loading, as reported in Figure 1.

From Figure 1, it was noticed that the increase in % enzyme loading from 1% to 10% affected the rate of conversion, while at 10% enzyme, the maximum conversion reached a maximum within a time of 15 min in both solution and bulk, compared to 1 and 2 h with 1% N435 in both bulk and diphenyl ether conditions, respectively. On the other hand, a temperature increase of 20 °C did not influence the reaction rate, but rather increased the maximum monomer conversion by a modest value of ~4%.

To validate the 2 h oligomerization step, a two-step polycondensation reaction was set up to run at constant temperature, % enzyme loading and vacuum. This test was set to determine if extending the oligomerization time would have any effect on the *M_n_* of the final product after a 24 h vacuum application. The corresponding conditions are enclosed in Table 2, where the only variable was the oligomerization time, followed by 24 h of vacuum application. The results confirm that, for an average value of 5.5 *w*/*w* of enzyme, there is no influence between 2 or 6 h oligomerization time where, e.g., the *M_n_* after 24 h were 6700, 7000 and 6700 g·mol^−1^ for 2, 4 and 6 h oligomerization, respectively. In other words, there is no need for prolongation, and an oligomerization step of 1 or 2 h is sufficient for further experiments.

Although the effect of temperature and catalyst loading were widely studied in the literature, the effect of vacuum variation was scarcely mentioned. The results obtained using the central composite design (CCD) of experiment did not only determine the effect of temperature, % enzyme loading and vacuum, but also the interactions between these factors. For solution polymerization, and following the method detailed under statistical analysis (Experimental section), a quadratic model was developed with a predicted *R*^2^ = 0.92. Further information on fit statistics for in solution polymerization is given in Table S2, the graph of the predicted vs. actual plots for in solution polymerization is given in Figure S8, and shows a very good fit of the data. The model is presented as an equation in terms of the actual factors given in Table S3.

After a 24 h reaction under vacuum, ^1^H NMR confirmed >90% conversion for all samples (sample results are provided in the supporting sheet Figures S6 and S7).

**Figure 2:**
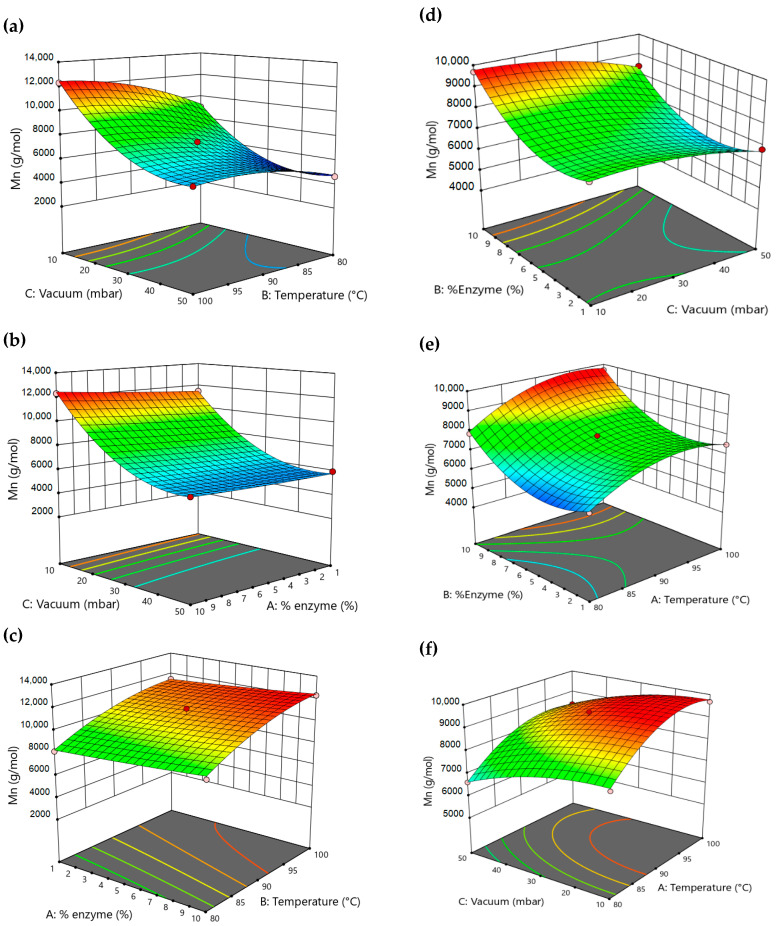
(**a**) Effect of vacuum and temperature on *M_n_* (solution) at 10% enzyme loading. (**b**) Effect of vacuum and % enzyme loading on *M_n_* (solution) at 100 °C. (**c**) Effect of % enzyme loading and temperature on *M_n_* (solution) at 10 mbar of reduced pressure. (**d**) Effect of vacuum and % loading enzyme on *M_n_* (bulk conditions) at 100 °C. (**e**) Effect of % enzyme loading and temperature on *M_n_* (bulk conditions) at 10 mbar of vacuum. (**f**) Effect of vacuum and temperature on *M_n_* (bulk conditions) at 10% enzyme loading. (The red and pink points represent experimental data above and below the predicted model, respectively).

As for the effect of the tested variables, the results clearly show that vacuum was the most influential factor on the *M_n_* achieved, where an increase from 50 to 10 mbar resulted in an increase of 4400 g·mol^−1^ at 80 °C and up to 6300 g·mol^−1^ at 100 °C, showing that vacuum becomes more influential at elevated temperatures. Similarly, the temperature increase had a pronounced effect on the *M_n_*, where an increase of 20 °C from 80 to 100 °C resulted in an increase in *M_n_* by 3300 g·mol^−1^ when the vacuum was 10 mbar. The effect of temperature did not have the same influence at the lower vacuum level of 50 mbar where the same temperature increase, resulted only in 1400 g·mol^−1^ increase in *M_n_* (see Figure 2a) increased with the increase in *M_n_*, with all values within a range of 1.2–1.7 (see Table S9). These results arise from a significant interaction between variables, which in this case is an interaction between temperature and vacuum. In other words, the effect of vacuum on *M_n_* is dependent on the level of temperature and *vice versa*. This vacuum-temperature interaction could be further justified by the Clausius-Clapeyron equation, where the vapor pressure of liquids increases with the increase in temperature in a non-linear manner [37,38], and thus, at higher temperatures, vacuum application becomes more efficient in the removal of ethanol due to a more pronounced increase in vapor pressure, thus pushing the reaction forward. Additionally, varying the % enzyme loading between 1% and 10% showed no significant effect on *M_n_* (see Figure 2b,c), suggesting that with 1% *w*/*w* N435, the catalyst exceeds the stoichiometric amount of reactive moieties present in the system and is therefore sufficient to catalyze the reaction.

Moving into bulk polymerization, and using the same variables and levels as in solution polymerization conditions to facilitate comparison, a quadratic model was designed with an *R*^2^ = 0.9. Further information on fit statistics for in bulk polymerization is given in Table S6, the graph of the predicted vs. actual plots for in bulk polymerization is given in Figure S9. The final equation in terms of actual factors for in bulk polymerization is given in Table S7. However, in contrary to the results in solution, the factors tested here had different influence on the *M_n_* of the synthesized polymer, where it was shown that enzyme loading had the most pronounced effect (see Figure 2d,e), followed by temperature, and finally vacuum (see Figure 2f) giving a less pronounced effect. Additionally, the *M_n_* achieved in bulk conditions was significantly lower than that achieved in solution following the same conditions. Dispersity (see Table S9) increased with *M_n_* but did not vary beyond the range of 1.2–1.6. These variations are not surprising. In fact, it could be explained by the decrease in diffusion capabilities of growing chains in high viscous mediums. In contrast to solution polymerization, bulk polymerization shows fast and significant increase in viscosity within minutes of vacuum application, leading to complete stop of stirring applied via magnetic bars. Having the catalyst in its heterogeneous form, it becomes more and more crucial to maintain adequate mass transfer to allow the polymer growth. In fact, though 1% N435 proved to be as efficient as 10% in solution, the results in bulk conditions showed significant variation between both percentages. This variation should be mainly attributed to the limitations enforced by the decrease in mass transfer rather than the activity of the enzyme, where a higher loading of N435 would rationally occupy more space within the medium, resulting in more interaction between substrates and the enzyme active sites especially when chain movement in the medium is reduced. Blank reactions (without enzyme) were performed in both solution and bulk conditions at 100 °C for 2 h oligomerization under atmospheric pressure, followed by 24 h under 10 mbar of vacuum. However, no precipitates were formed with cold methanol, suggesting that no polymer growth was achieved without N435.

**Figure 3:**
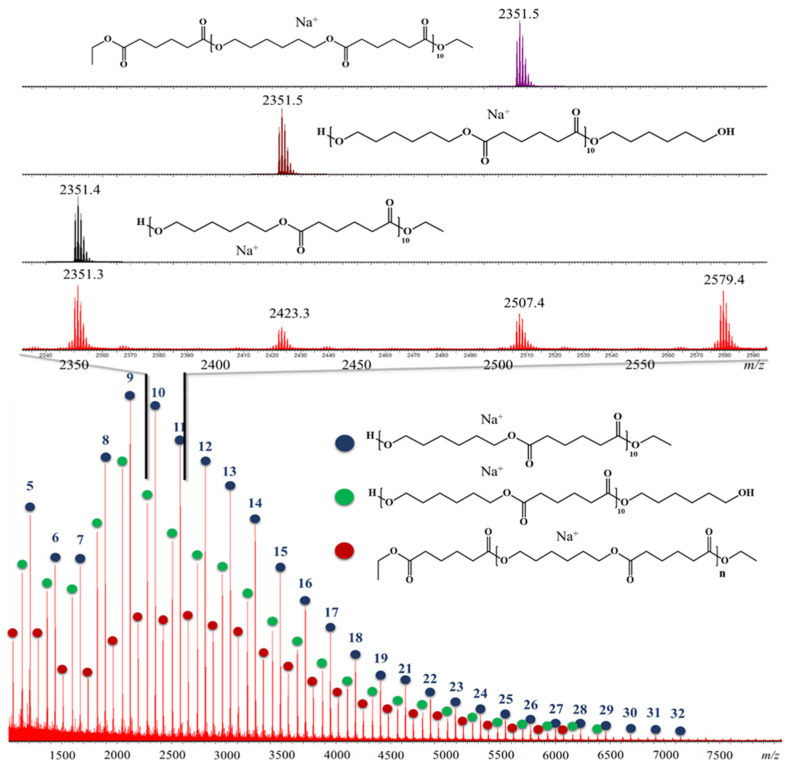
MALDI mass spectrum recorded for experiment 1S, lower part of the figure represents the global mass spectrum with the 3 main families present in the polymer sample, the number on each signal highlights the number of monomer units for the observed ions. The upper part of the spectrum corresponds to a magnification between *m*/*z* 2300 and *m*/*z* 2600 with a comparison between the theoretical isotopic models (for oligomers with 10 monomer units of diol and diester) and the experimental data confirming the presence of 3 end-groups moieties, i.e., ester-ester (red dots), alcohol-alcohol (green dots) and ester-alcohol (blue dots), respectively.

MALDI-ToF MS was used to determine the nature of the end-groups present in the synthesized poly(hexylene adipate). However, due to the quite broad molar mass dispersity (i.e., >1.2) of the polymer samples, mass spectrometry was not useful for determining the molar masses accurately [39]. As such, 16 polymer samples (8 solution and 8 bulk conditions) were analyzed to establish a comparison between both conditions as represented in Table S9. From the MS spectra in Figure 3 representing experiment 1S, three main polyester families were identified being end-functionalized as (1) ester-ester, (2) alcohol-alcohol, and (3) ester-alcohol. Cyclic structure was also probable but only traces were detected. Based on the MALDI spectrum, ester-alcohol was found to possess the highest intensity, showing that these structures are the most abundant in the sample, as expected. Those results were consistent among all the tested samples in bulk and solution media with no apparent differences.

**Figure 4:**
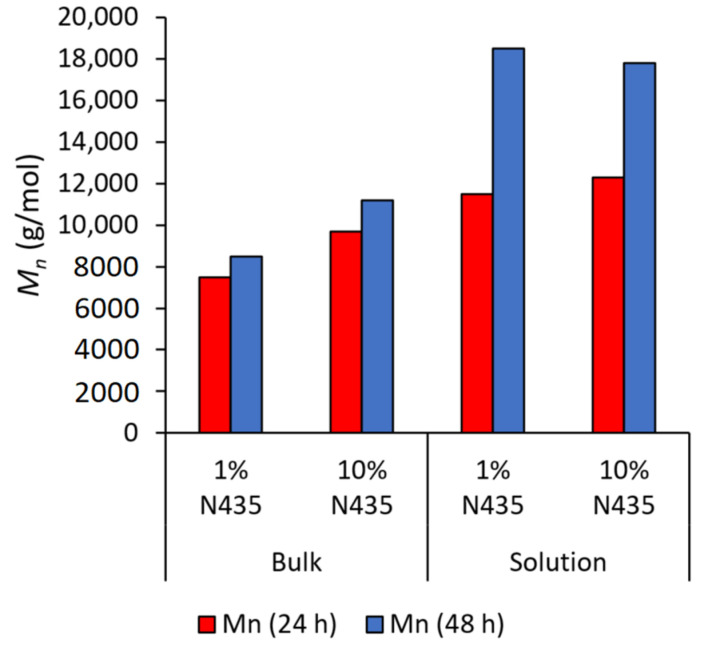
Effect of extending polymerization time on *M_n_* in both solution and bulk conditions.

The limitation imposed by the decrease in mass transfer becomes more apparent when extending the reaction time (see Figure 4), where the *M_n_* of polyesters synthesized in bulk increase only by 30% when extending the reaction time from 24 to 48 h. On the other hand, up to 70% increase in *M_n_* was observed in solution, mainly due to both better mixing and mass transfer. The high positive influence of efficient mixing was previously highlighted using reactive extrusion for the ring-opening polymerization of ω-pentadecalactone, yielding an *M_n_* of 90,000 g·mol^−1^ in only 15 min compared to 22,100 g·mol^−1^ after 72 h in bulk conditions [40]. In this work, the *M_n_* of the first cycle in both mediums was considered as 100%, while the percentage yield was calculated by dividing the actual yield by the theoretical yield, taking into consideration the molar mass of the polyester achieved.

**Figure 5:**
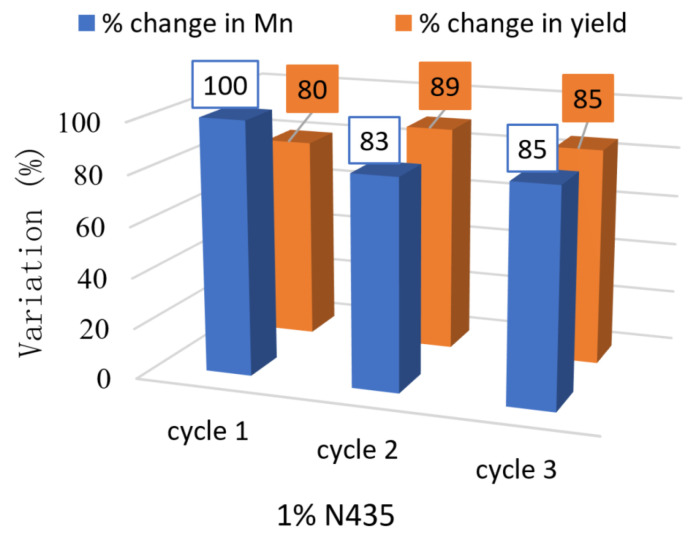
Effect of recycling 1% N435 on *M_n_* and yield in solution.

**Figure 6:**
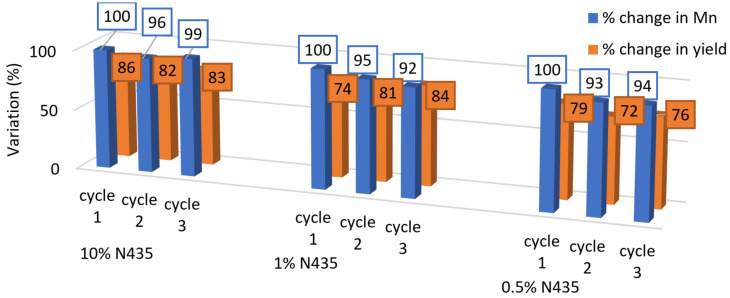
Effect of recycling 0.5%, 1% and 10% N435 on *M_n_* and yield in bulk conditions.

The recyclability of N435 was finally tested for three consecutive cycles in solution (1% *w*/*w* N435) (see Figure 5) and bulk conditions (0.5%, 1% and 10% *w*/*w* N435) (see Figure 6). The results for solution polycondensation showed ~17% drop in *M_n_* during the second cycle from 12,100 to 10,000 g·mol^−1^; however, no significant changes were observed during the third cycle.

On the other hand, the recyclability assays in bulk conditions showed better consistency during the three cycles, where the *M_n_* dropped by a maximum of 8% in the third cycle with 1% N435, and to a lower extent with 0.5% and 10% N435, knowing that it was considered insignificant as it falls within the error range of the GPC analysis. As such, for bulk polymerization, even at relatively high temperature (100 °C), N435 can be effectively reused at least for three consecutive cycles giving similar results in terms of *M_n_* and yield as observed in Figure 6. The more pronounced drop in N435 activity in diphenyl ether medium can be attributed to several reasons. First, the use of diphenyl ether as a solvent attributed to a better heat transfer in the system, and thus, the enzyme will be more prone to elevated temperatures in comparison to bulk, which would result in a more pronounced enzyme degradation or leaching. Additionally, due to the fact that N435 is prepared via interfacial activation of lipases vs. supports with hydrophobic surfaces, the enzyme becomes more susceptible to be released in the presence of organic solvents [17,41,42]. Moreover, the activity of enzymes can also be influenced by the water activity, Secundo et al. showed a drop in the transesterification activity for the reaction between vinylacetate and 1-octanol as a function of water activity in different formulations of CALB, including N435 [43]. Similarly, other works found a similar relation between the increase in water activity and the decrease in enzyme activity and maximum achieved *M_n_* [44,45,46,47].

However, this remains speculation within the current study and further studies are needed to determine the reason behind the drop in N435 activity in solution.

## 4. Conclusions

The polycondensation reaction of 1,6-hexanediol and diethyl adipate was studied in both diphenyl ether and bulk media using Novozym 435 as biocatalyst. The oligomerization time was optimized where the maximum monomer conversion was confirmed after a maximum of two hours from the start of the reaction, thus preventing any unnecessary time extension for the oligomerization step. Following this, a face-centered central composite design was used to develop a quadratic model that showed how temperature, vacuum and % enzyme loading can affect *M_n_*. In diphenyl ether, vacuum showed to be the most influential factor in relation to *M_n_*, followed by temperature and finally % enzyme loading that showed no significant effect. This relation between the independent variables and *M_n_* showed an opposite relation in bulk, where % enzyme loading had the most significant impact on *M_n_*, followed by temperature and vacuum, respectively. The models were confirmed by running additional experiments, where their results were within the acceptable prediction interval, confirming that the models can be adequately used to predict the *M_n_* of the polymer at any level within the tested factor ranges. Recyclability assays showed a more efficient recycling for N435 in bulk conditions (consistent results up to three cycles) in comparison to diphenyl ether that showed a drop of activity by 17% for the second cycle. Finally, this work introduced a green method to produce poly(hexylene adipate) and control its *M_n_*_._ Future research will enlarge on the findings of this work, to develop efficient processes that would overcome some of the major limitations encountered herein, such as low heat and mass transfer that limit polymer growth. Enzymatic reactive extrusion is currently under investigation by our team as a powerful tool employed to surpass the aforementioned obstacles and transform traditional batch production processes into more dynamic continuous processes that can achieve high molecular weights within short periods of time.

## Figures and Tables

**Table 1:** Effect of monomer concentration on yield, *M_n_* and dispersity of poly(hexylene adipate).

Entry ^1^	Concentration (mol·L^−1^)	Yield (%)	*M_n_* (g·mol^−1^) ^2^	Đ_M_^3^
**1**	4	88	12,300	1.44
**2**	2	86	10,700	1.49
**3**	1	74	8300	1.30
**4**	0.5	56	7400	1.18

^1^ All experiments were conducted following a two-step polycondensation reaction: 1st step reaction conducted under atmospheric pressure, followed by the 2nd step of 24 h under vacuum (10 mbar). Temperature and enzyme loading were kept constant in both steps at 100 °C and 10% *w*/*w* of N435. ^2^ The number average molecular weight (*M_n_*) was obtained from GPC analyses (CHCl_3_, 40 °C, polystyrene standards). ^3^ Molar mass dispersity Đ_M_ = *M_W_*/*M_n_* was obtained from GPC analyses (CHCl_3_, 40 °C, polystyrene standards).

**Table 2:** Effect of oligomerization time variation on yield, *M_n_* and dispersity after a 24 h (secondary step) under vacuum.

Entry	Reaction Time (h) ^1^ 1st Step/2nd Step	Yield (%)	*M_n_*(g·mol^−1^) ^2^	Đ_M_^3^
**5**	2/24	88	6700	1.38
**6**	4/24	86	7000	1.34
**7**	6/24	74	6700	1.38

^1^ 1st step reaction conducted under atmospheric pressure, followed by the 2nd step of 24 h under vacuum (30 mbar). Temperature and enzyme loading were kept constant in both steps at 90 °C and 5.5% *w*/*w* of N435. ^2^ The number average molecular weight (*M_n_*) was obtained from GPC analyses (CHCl_3_, 40 °C, polystyrene standards). ^3^ Molar mass dispersity Đ_M_ = *M_W_*/*M_n_* was obtained from GPC analyses (CHCl_3_, 40 °C, polystyrene standards).

## Supplementary Materials

The following are available online at https://www.mdpi.com/2073-4360/12/9/1907/s1, Figure S1: 1,6-hexanediol (C_6_H_14_O_2_) ^1^H NMR spectrum (CDCl_3_, 300 MHz): *δ* 1.39 (m, 4H), 1.58 (m, 4H), 3.64 (t, *J* = 6.5 Hz, 4H); Figure S2: Diethyl adipate (C_10_H_18_O_4_) ^1^H NMR spectrum (CDCl_3_, 300 MHz): *δ* 1.25 (t, *J* = 7.1 Hz, 6H), 1.66 (m, 4H), 2.31 (t, *J* = 7.2 Hz, 4H), 4.11–4.13 (q, *J* = 7.1 Hz, 4H); Figure S3: Poly(hexylene adipate) [–O(CH_2_)_6_O_2_C(CH_2_)_4_CO–]_n_^1^H NMR spectrum (CDCl_3_, 300 MHz): *δ* 1.37 (m, 4H), 1.66 (m, 8H), 2.32 (t, *J* = 7.1 Hz, 4H), 4.06 (t, *J* = 6.7 Hz, 4H); Figure S4: ^1^H NMR spectrum (CDCl_3_, 300 MHz) of the crude reaction of 1,6-hexanediol and diethyl adipate in diphenyl ether (1 mL), and the yielded Poly(hexylene adipate) after 15 min reaction at 80 °C and 1% *w*/*w* enzyme loading: *δ* 1.25 (t, 6H), 1.37 (m, 8H), 1.66 (m, 12H), 2.32 (t, 8H), 3.65 (t, 4H), 4.06 (t, 4H), 4.11–4.13 (q, 4H). *Note: δ ~7–7.5 represent diphenyl ether;* Figure S5: Enlarged view of figure S4: (between 2.2 and 4.2 ppm); Figure S6: ^1^H NMR spectrum (CDCl_3_, 300 MHz) of the crude reaction of 1,6-hexanediol and diethyl adipate in bulk, and the yielded Poly(hexylene adipate) after 24 h at 50 mbar vacuum application, at 90 °C and 5.5% *w*/*w* enzyme loading; Figure S7: ^1^H NMR spectrum (CDCl_3_, 300 MHz) of the crude reaction of 1,6-hexanediol and diethyl adipate in 1 mL diphenyl ether, and the yielded Poly(hexylene adipate) after 24 h at 10 mbar vacuum application, at 100 °C and 1% *w*/*w* enzyme loading. *Note: δ ~7–7.5 represent diphenyl ether;* Figure S8: Graph of the predicted vs. actual plots in solution polymerization; Figure S9: Graph of the predicted vs. actual plots in bulk polymerization; Table S1: Build information of the design model for in solution polymerization; Table S2: Fit statistics for in solution polymerization; Table S3: Final equation in term of actual factors (in-solution polymerization); Table S4: Additional tested point for model confirmation for in solution polymerization; Table S5: Build information of the design model for bulk polymerization; Table S6: Fit statistics for bulk polymerization; Table S7: Final equation in term of actual factors (bulk polymerization); Table S8: Additional tested point for model confirmation for in solution polymerization; Table S9: Experiments analyzed via MALDI-TOF MS for end group determination.
