# Supplementary material for: Enzymatic Polycondensation of 1,6-Hexanediol and Diethyl Adipate: A Statistical Approach Predicting the Key-Parameters in Solution and in Bulk
Source: Polymers (Basel). 2020 Aug 24;12(9):1907. doi: 10.3390/polym12091907 (PMC7565462; doi:10.3390/polym12091907)
Supplement: Supplementary file 1 [file polymers-12-01907-s001.pdf]

Supporting information

Enzymatic polycondensation of 1,6-hexanediol and diethyl adipate: A statistical approach predicting the key-parameters in solution and in bulk

Kifah Nasr[1,2], Julie Meimoun[1], Audrey Favrelle-Huret[1], Julien De Winter[3], Jean-Marie Raquez[2*], Philippe Zinck[1*]

Contents

^{1}H NMR spectra: 2

Statistical information (in solution polymerization) 9

Statistical information (bulk polymerization) 11

MALDI-TOF MS tested samples 15

^{1}H NMR spectra:

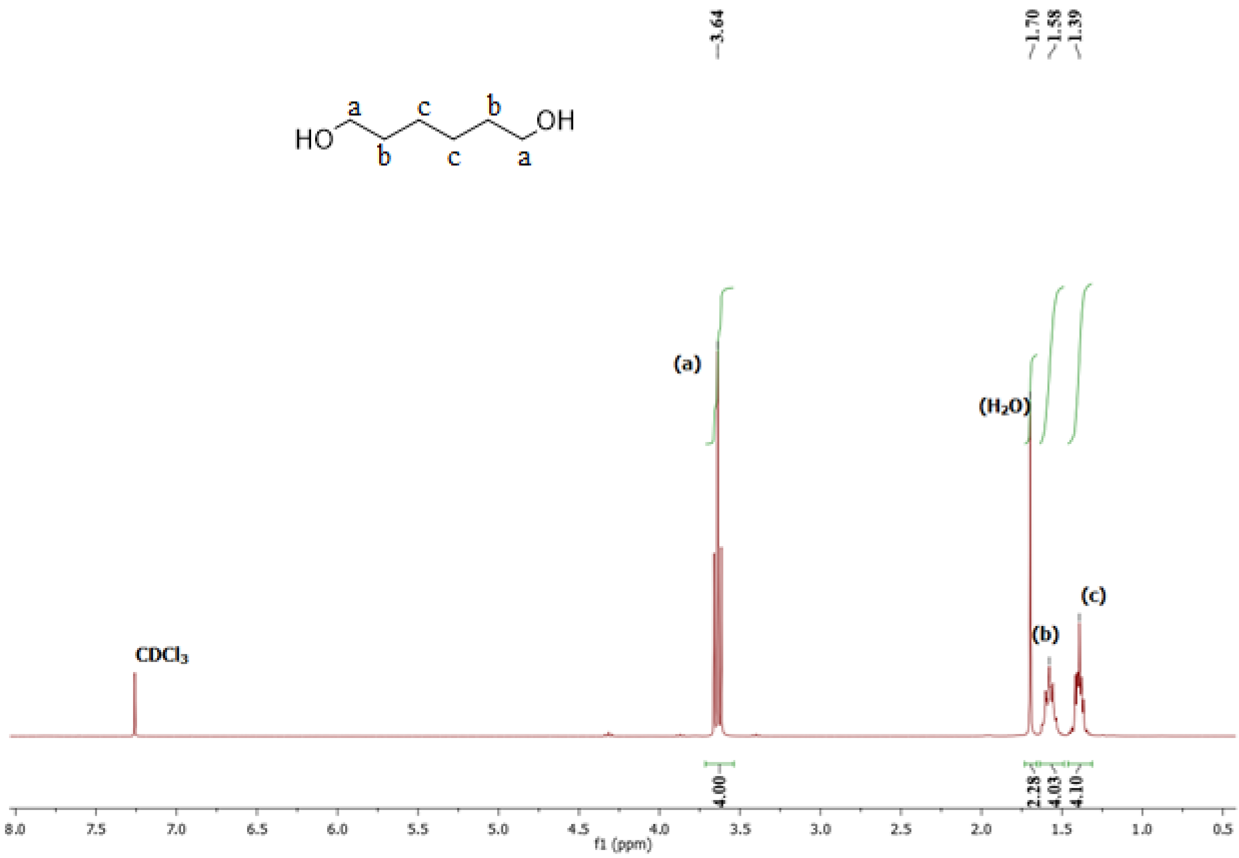

Figure S1: 1,6-hexanediol ($C_6H_{14}O_2$) ^{1}H NMR spectrum ($CDCl_3$, 300 MHz): δ 1.39 (m, 4H), 1.58 (m, 4H), 3.64 (t, J = 6.5 Hz, 4H).

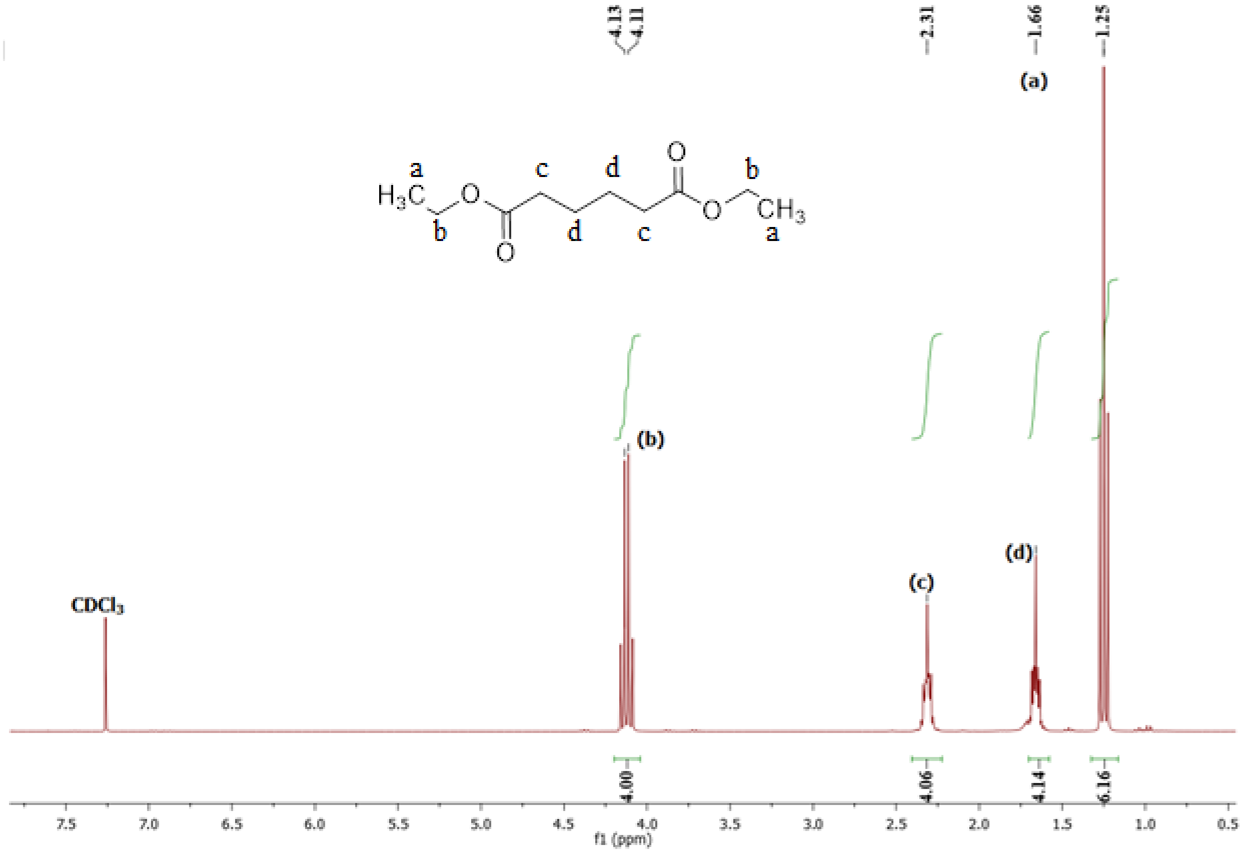

Figure S2: Diethyl adipate ($C_{10}H_{18}O_4$) ^{1}H NMR spectrum ($CDCl_3$, 300 MHz): δ 1.25 (t, *J* = 7.1 Hz, 6H), 1.66 (m, 4H), 2.31 (t, *J* = 7.2 Hz, 4H), 4.11-4.13 (q, *J* = 7.1 Hz, 4H).

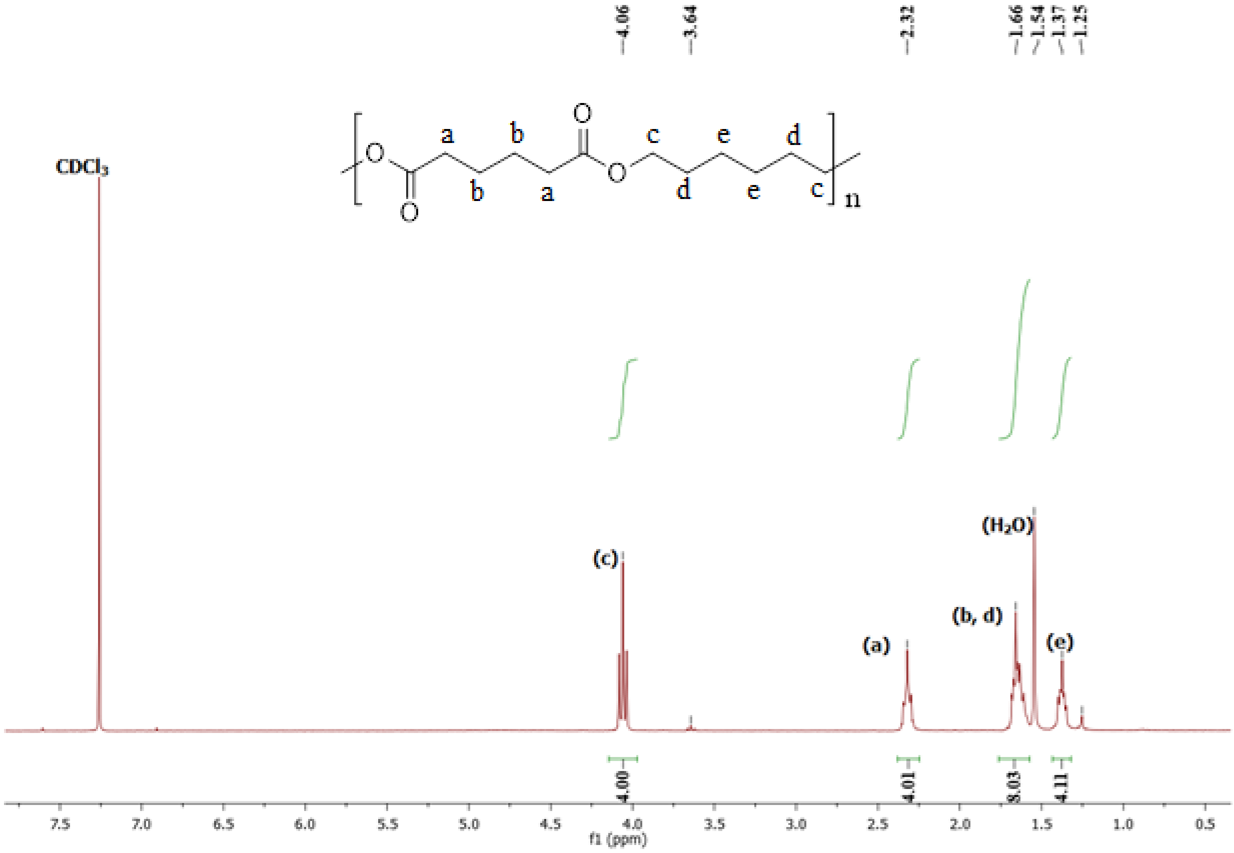

Figure S3: Poly(hexylene adipate) [-O(CH_2)$_6$$O_2$C($CH_2$)$_4$CO-]n ^{1}H NMR spectrum ($CDCl_3$, 300 MHz): δ 1.37 (m, 4H), 1.66 (m, 8H), 2.32 (t, *J* = 7.1 Hz, 4H), 4.06 (t, *J* = 6.7 Hz, 4H).

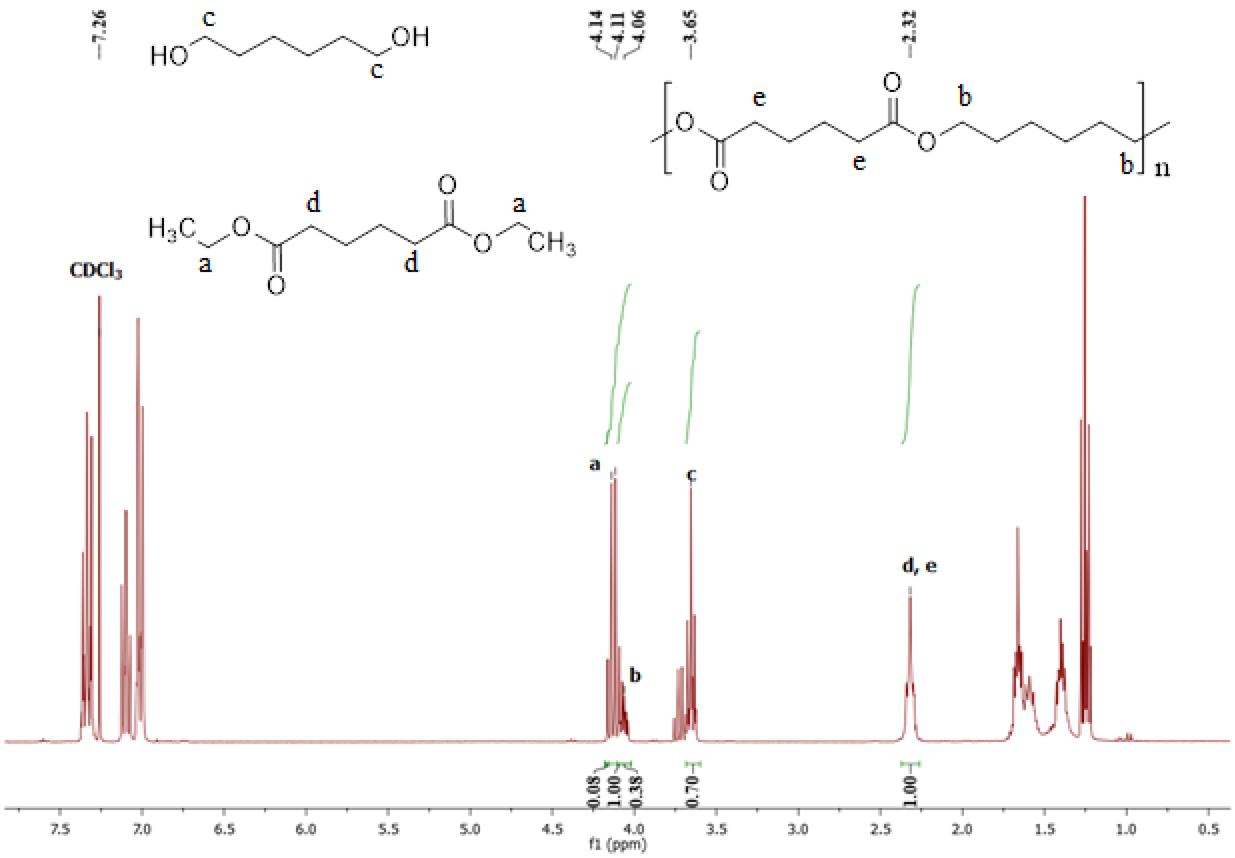

Figure S4: ^{1}H NMR spectrum ($CDCl_3$, 300 MHz) of the crude reaction of 1,6-hexanediol and diethyl adipate in diphenyl ether (1 mL), and the yielded Poly(hexylene adipate) after 15 mins reaction at 80 °C and 1% w/w enzyme loading: δ 1.25 (t, 6H), 1.37 (m, 8H), 1.66 (m, 12H), 2.32 (t, 8H), 3.65 (t, 4H), 4.06 (t, 4H), 4.11-4.13 (q, 4H). *Note: δ ~7-7.5 represent diphenyl ether.*

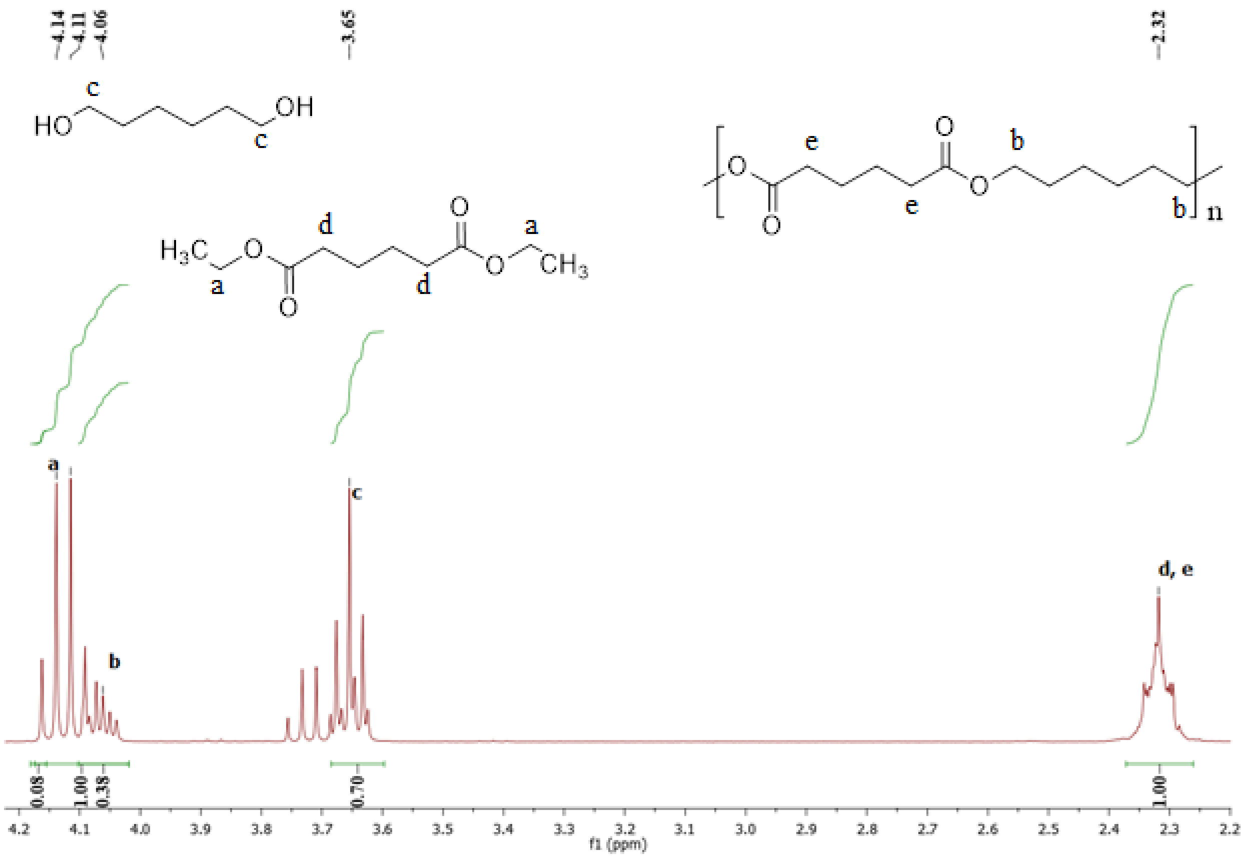

Figure S5: Enlarged view of figure S4 (between 2.2 and 4.2 ppm).

The totality of the unreacted monomer in addition to the produced polymer was presented as an integral of 1 that can be calculated via two ways, either by considering the integral for Poly(hexylene adipate) and unreacted diethyl adipate (-OOC-$\underline{CH_2}$-CH_2-CH_2-$\underline{CH_2}$-COO-) represented at δ= 2.32 or by considering the combination of signals at δ= 4.11-4.14 of the methylene group (CH_3-$\underline{CH_2}$-O-) of the unreacted diethyl adipate in addition to the signals at δ= 4.06 of the methylene (-O-$\underline{CH_2}$-C_4H_8-$\underline{CH_2}$-O-) of the produced Poly(hexylene adipate). The conversion is considered as the ratio of the integral presenting the methylene (-O-$\underline{CH_2}$-C_4H_8-$\underline{CH_2}$-O-) of the produced Poly(hexylene adipate) to the total mentioned above given in Equation S1: $Conversion = 100 \times \frac{I_b}{I_a+I_b}$, where I_b is the integral of methylene (-O-$\underline{CH_2}$-C_4H_8-$\underline{CH_2}$-O-) of the produced Poly(hexylene adipate), and I_a is the integral of the methylene group (CH_3-$\underline{CH_2}$-O-) of the unreacted diethyl adipate. Due to partial peak overlapping between the triplets representing the methylene (-O-$\underline{CH_2}$-C_4H_8-$\underline{CH_2}$-O-) of Poly(hexylene adipate) at δ= 4.06 and the right peak of the

quartet representing the unreacted diethyl adipate (-OOC-CH2-CH2-CH2-CH2-COO-) at δ= 2.32, the conversion was calculated by including the mentioned peak as an integral of Poly(hexylene adipate) and subtracting its value represented by the left peak of the quartet (0.38-0.08= 0.3= 30% conversion).

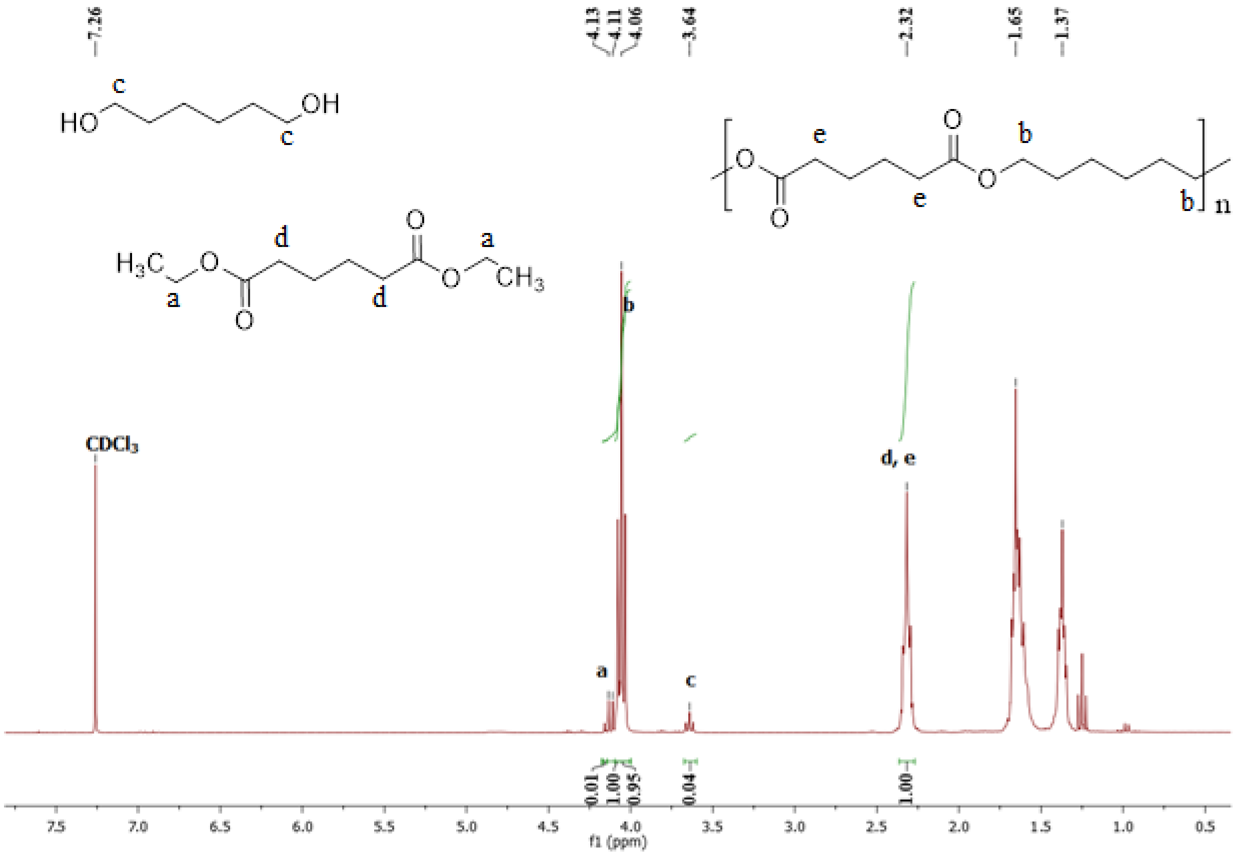

Figure S6: ^1H NMR spectrum ($CDCl_3$, 300 MHz) of the crude reaction of 1,6-hexanediol and diethyl adipate in bulk, and the yielded Poly(hexylene adipate) after 24 h at 50 mbar vacuum application, at 90 °C and 5.5% w/w enzyme loading.

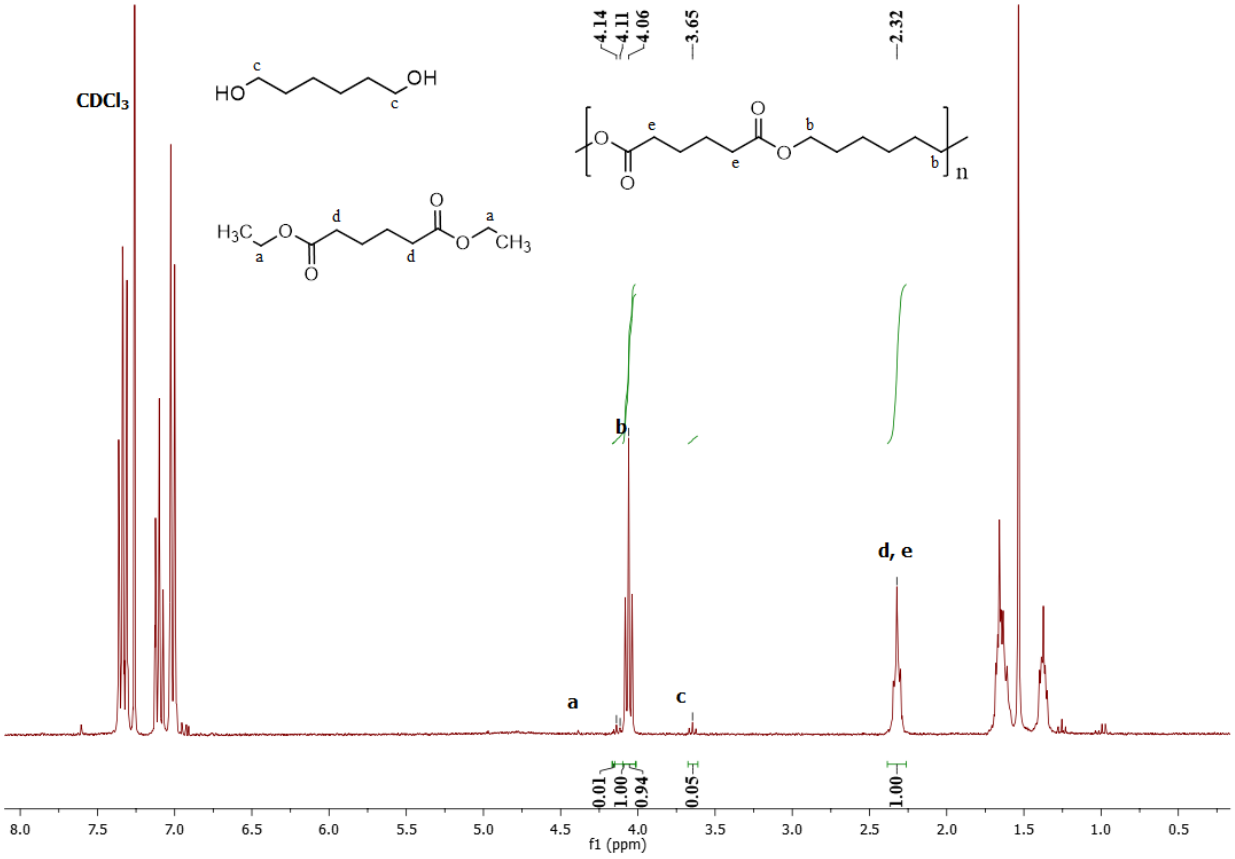

Figure S7: ^{1}H NMR spectrum ($CDCl_3$, 300 MHz) of the crude reaction of 1,6-hexanediol and diethyl adipate in 1 mL diphenyl ether, and the yielded Poly(hexylene adipate) after 24 h at 10 mbar vacuum application, at 100 °C and 1% w/w enzyme loading. *Note: δ ~7-7.5 represent diphenyl ether.*

Statistical information (in solution polymerization)
Build Information

Table S1: Build information of the design model for in solution polymerization.

**File Version**	11.1.2.0			
**Study Type**	Response Surface		**Subtype**	Randomized
**Design Type**	I-optimal	Coordinate Exchange	**Runs**	18
**Design Model**	Quadratic		**Blocks**	No Blocks

Fit Statistics

Table S2: Fit statistics for in solution polymerization.

**Std. Dev.**	**415.60**	**R^2**	**0.9851**
**Mean**	7288.56	**Adjusted R^2**	0.9683
**C.V. %**	5.70	**Predicted R^2**	0.9231
		**Adeq Precision**	25.5240

The **Predicted R^2** of 0.9231 is in reasonable agreement with the **Adjusted R^2** of 0.9683; *i.e.* the difference is less than 0.2.

Adeq Precision measures the signal to noise ratio. A ratio greater than 4 is desirable. The ratio of 25.524 indicates an adequate signal. This model can be used to navigate the design space.

Table S3: Final equation in term of actual factors (in-solution polymerization).

M_n	=
**-57746.63612**	
**+102.81721**	% w/w enzyme loading
**+1409.90006**	Temperature
**-170.83302**	Vacuum
**-0.091667**	% enzyme * Temperature
**-2.09306**	% w/w enzyme loading * Vacuum
**-2.38562**	Temperature * Vacuum
**+3.05879**	% w/w enzyme loading^2
**-6.82060**	Temperature^2
**+4.48610**	Vacuum^2

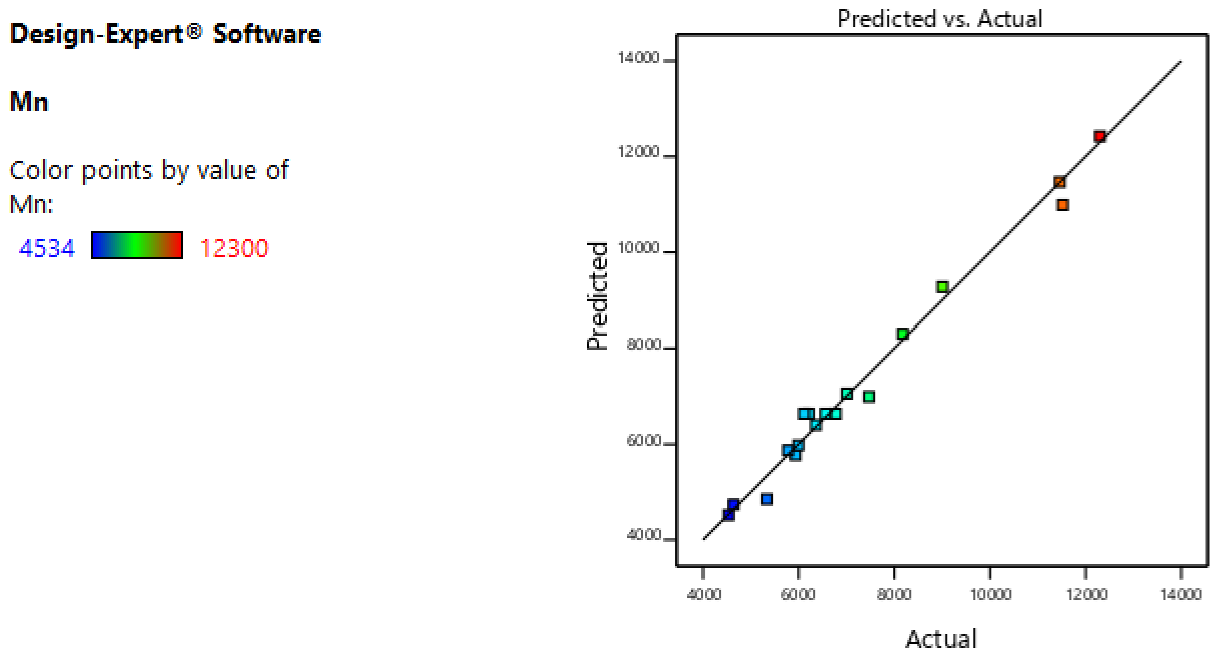

Figure S8: Graph of the predicted *vs.* actual plots in solution polymerization.

Confirmation

Two-sided Confidence = 95%

Table S4: Additional tested point for model confirmation for in solution polymerization.

Temperature (°C)	% w/w enzyme loading	Vacuum (mbar)	Response	Predicted Mean	Predicted Median	Std Dev	n	SE Pred	95% PI low	Data Mean	95% PI high
**90**	10	50	Mn	6040.86	6040.86	415.602	1	544.717	4784.74	5955	7296.98
**100**	5.5	50	Mn	5814.91	5814.91	415.602	1	544.717	4558.79	6699	7071.03
**90**	10	10	Mn	11533	11533	415.602	1	544.717	10276.9	10340	12789.1
**100**	5.5	10	Mn	11884.6	11884.6	415.602	1	544.717	10628.4	11300	13140.7
**100**	1	10	Mn	11467.9	11467.9	415.602	2	472.773	10377.6	12250	12558.1

Statistical information (bulk polymerization)
Build Information

Table S5: Build information of the design model for bulk polymerization.

**File Version**	11.1.2.0		
**Study Type**	Response Surface	**Subtype**	Randomized
**Design Type**	Central Composite	**Runs**	18
**Design Model**	Quadratic	**Blocks**	No Blocks

Fit Statistics

Table S6: Fit statistics for bulk polymerization.

**Std. Dev.**	**292.34**	**R^2**	**0.9697**
**Mean**	7290.78	**Adjusted R^2**	0.9355
**C.V. %**	4.01	**Predicted R^2**	0.9000
		**Adeq Precision**	18.9389

The **Predicted R^2** of 0.9000 is in reasonable agreement with the **Adjusted R^2** of 0.9355; *i.e.* the difference is less than 0.2.

Adeq Precision measures the signal to noise ratio. A ratio greater than 4 is desirable. The ratio of 18.939 indicates an adequate signal. This model can be used to navigate the design space.

Table S7: Final equation in term of actual factors (bulk polymerization).

M_n	=
**-61780.81204**	
**+1489.13946**	Temperature
**-674.83906**	% w/w enzyme loading
**+43.99298**	Vacuum
**+3.38892**	Temperature * % w/w enzyme loading
**-0.090008**	Temperature * Vacuum
**-1.79724**	% w/w enzyme loading * Vacuum
**-7.95682**	$Temperature^2$
**+54.80580**	% w/w enzyme $loading^2$
**-0.874206**	$Vacuum^2$

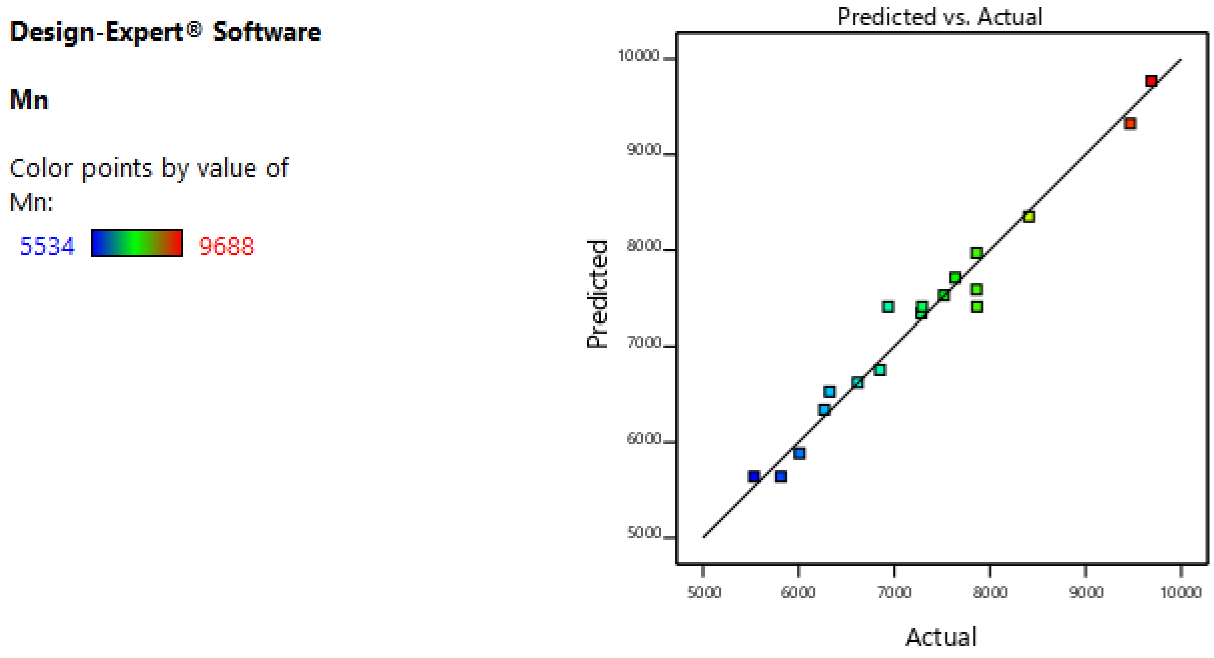

Figure S9: Graph of the predicted *vs.* actual plots in bulk polymerization.

Confirmation

Two-sided Confidence = 95%

Table S8: Additional tested point for model confirmation for in solution polymerization.

Temperature (°C)	% w/w enzyme loading	Vacuum (mbar)	Response	Predicted Mean	Predicted Median	Std Dev	n	SE Pred	95% PI low	Data Mean	95% PI high
**100**	1	10	Mn	7528.28	7528.28	292.343	1	390.969	6626.7	7039	8429.85
**90**	1	10	Mn	7729.96	7729.96	292.343	1	383.149	6846.42	7065	8613.5
**90**	5.5	10	Mn	7587.89	7587.89	292.343	1	359.478	6758.93	8221	8416.85
**80**	10	50	Mn	6625.48	6625.48	292.343	1	391.098	5723.6	5832	7527.35
**90**	5.5	50	Mn	6530.09	6530.09	292.343	2	293.411	5853.49	6105.5	7206.7
**80**	10	10	Mn	7970.78	7970.78	292.343	1	390.969	7069.2	7678	8872.35
**100**	10	10	Mn	9768.78	9768.78	292.343	1	389.332	8870.98	9525	10666.6

MALDI-TOF MS tested samples

Table S9: Experiments analyzed via MALDI-TOF MS for end group determination.

			Bulk polycondensation				In-solution polycondensation			
**Experimental conditions**				**Determined via SEC**				**Determined via SEC**		
Enzyme	***Temperature***	***Vacuum***		***Mn***	***Mw***	***dispersity***		***Mn***	***Mw***	***dispersity***
**% w/w**	**°C**	**mbar**	**Experiment**	**g/mol**	**g/mol**		**Experiment**	**g/mol**	**g/mol**	
**1**	80	50	**1B**	5500	6800	1.2	**1S**	4500	5900	1.3
**10**	80	50	**2B**	6600	8200	1.2	**2S**	4600	6000	1.3
**1**	100	50	**3B**	6900	9100	1.3	**3S**	5900	7700	1.3
**10**	100	50	**4B**	8400	10700	1.3	**4S**	6000	8200	1.4
**1**	80	10	**5B**	6300	8400	1.3	**5S**	8200	11800	1.5
**10**	80	10	**6B**	7900	11200	1.4	**6S**	9000	13300	1.5
**1**	100	10	**7B**	7500	10100	1.3	**7S**	11500	17800	1.6
**10**	100	10	**8B**	9700	15700	1.6	**8S**	12300	19400	1.6
